# Targeting Human Parainfluenza Virus Type-1 Haemagglutinin-Neuraminidase with Mechanism-Based Inhibitors

**DOI:** 10.3390/v11050417

**Published:** 2019-05-05

**Authors:** Tanguy Eveno, Larissa Dirr, Ibrahim M. El-Deeb, Patrice Guillon, Mark von Itzstein

**Affiliations:** Institute for Glycomics, Griffith University, Gold Coast Campus, Queensland 4222, Australia; Tanguy.eveno@griffithuni.edu.au (T.E.); L.dirr@griffith.edu.au (L.D.); I.el-deeb@griffith.edu.au (I.M.E.-D.); p.guillon@griffith.edu.au (P.G.)

**Keywords:** parainfluenza, haemagglutinin, neuraminidase, difluorosialic acid, glycohydrolase, sialidase, inhibitor

## Abstract

Human parainfluenza virus (hPIV) infections are a major cause of respiratory tract illnesses in children, with currently no available vaccine or drug treatment. The surface glycoprotein haemagglutinin-neuraminidase (HN) of hPIV has a central role in the viral life cycle, including neuraminic acid-recognising receptor binding activity (early stage) and receptor-destroying activity (late stage), which makes it an ideal target for antiviral drug disovery. In this study, we showed that targeting the catalytic mechanism of hPIV-1 HN by a 2α,3β-difluoro derivative of the known hPIV-1 inhibitor, BCX 2798, produced more potent inhibition of the neuraminidase function which is reflected by a stronger inhibition of viral replication. The difluorosialic acid-based inhibitor efficiently blocked the neuraminidase activity of HN for a prolonged period of time relative to its unsaturated neuraminic acid (Neu2en) analogue, BCX 2798 and produced a more efficient inhibition of the HN neuraminidase activity as well as in vitro viral replication. This prolonged inhibition of the hPIV-1 HN protein suggests covalent binding of the inhibitor to a key catalytic amino acid, making this compound a new lead for a novel class of more potent hPIV-1 mechanism-based inhibitors.

## 1. Introduction

Viral infections of the respiratory tract are a major threat to the health of children, the immunocompromised, and the elderly [1]. Viruses form the family *Paramyxoviridae* and particularly human parainfluenza viruses type 1 (hPIV-1) and type 3 (hPIV-3) are common causes for moderate to severe respiratory tract illnesses [2]. hPIV-1 is generally associated with laryngotracheobronchitis (also known as croup), whereas hPIV-3 is predominantly responsible for bronchitis, bronchiolitis, and pneumonia, with both viruses causing significant economic burden [3,4]. Despite ongoing efforts [5,6], there is currently neither a vaccine nor a drug available to protect against or treat hPIV infections, respectively.

**Figure 1:**
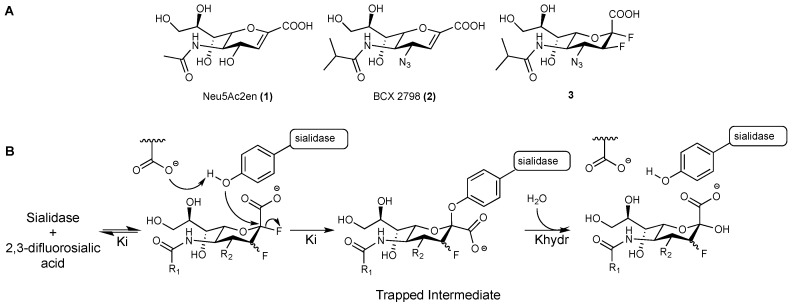
(**A**). Structures of Neu5Ac2en (**1**), BCX 2798 (**2**), and the difluoro analogue of BCX 2798 (**3**); (**B**). Proposed catalytic mechanism for all of the studied sialidases confirmed by the X-ray crystal structures of the trapped neuraminosyl–enzyme intermediate.

The hPIV surface glycoprotein haemagglutinin-neuraminidase (HN) is involved in the early stages of the virus replication cycle by mediating the binding to neuraminic acid-containing receptors through its haemagglutinin function, and triggering the fusion of the virus envelope with the host cell membrane. It is also responsible for the release of the virion progeny in the later stages of the viral life cycle through its neuraminidase activity, a highly specialised sialidase function that is responsible for the cleavage of terminal neuraminic acid from sialoglycoconjugates [2]. The multifunctionality of hPIV HN makes it a target of choice for the design of antiviral drugs. Several inhibitors based on the 2-deoxy-2,3-didehydro-D-*N*-acetylneuraminic acid (Neu5Ac2en, **1**) scaffold, such as BCX 2798 (**2**) [7,8] (Figure 1A), have been designed to target hPIV-1 HN’s catalytic domain, but none have advanced to clinical studies [9,10,11,12,13].

Recently, a number of studies on sialidases from different organisms, including human Neu2 [14], *Trypanosoma cruzi* (*T. cruzi*) trans-sialidase [15], influenza A virus (IAV) neuraminidase (NA) [16], and human parainfluenza virus type 3 (hPIV-3) HN [17], have revealed that they all share a common catalytic mechanism. These sialidases were found to act through a two-step, double-displacement mechanism, where a highly-conserved tyrosine residue in the sialic acid-binding pocket is involved as a catalytic nucleophile to form a covalent bond with the C-2 of the bound sialic acid moiety (Figure 1B). In order to prove such a catalytic mechanism, various 2,3-difluorosialic acid derivatives have been used as probes. The α-fluorine at the anomeric position serves as an excellent leaving group that accelerates the formation of the covalent tyrosinyl–neuraminosyl intermediate, while the fluorine at C-3 inductively stabilises the oxocarbenium ion-like transition state to reduce the rates of both glycosylation and deglycosylation, and consequently increases the half-life of the trapped neuraminosyl–enzyme intermediate [14,16]. The prolonged binding combined with the potent inhibition exerted by these probes have made them a promising novel class of sialidase inhibitors, with the potential to be developed into therapeutics [16,17].

2β,3β-difluorosialic acid derivatives have been previously evaluated against hPIV-1 HN but failed to show sub-millimolar neuraminidase inhibiton [18], probably because of the unfavourable stereoconfiguration of the installed fluoride at C-2. Accordingly, in this study we have used a different 2,3-difluorosialic acid probe, the 2α,3β-difluoro analogue of BCX 2798 (**3**), to investigate its inhibitory profile against hPIV-1 HN’s functions and evaluate its capacity to act as a mechanistic inhibitor for viral replication. We have recently reported the use of this probe to explore the catalytic mechanism of hPIV-3 HN [17]. The compound was also found to act as a mechanism-based inhibitor by forming a covalent intermediate with the catalytic residue Tyr530 of hPIV-3 HN, resulting in a prolonged inhibitory effect. Herein, the potential of the 2α,3β-difluoro derivative of BCX 2798 (**3**) to block the catalytic activity of hPIV-1 HN has been defined using ^1^H NMR spectroscopy. The impact of this blockade on the inhibition of hPIV-1 HN’s functions and consequently on viral replication has been evaluated with appropriate virological methods.

## 2. Material and Methods

### 2.1. hPIV-1 HN Inhibitors

Compounds **2** and **3** were synthesised according to reported procedures [17,19] and were provided as lyophilised powder. Initial stocks were resuspended in either sterile MilliQ H_2_O at 40 mM or in D_2_O at 50 mM and further dilutions were made in the appropriate reaction buffer.

### 2.2. Cells and Viruses

MA104 cells were maintained at 37 °C, 5% CO_2_ in Eagle’s Minimum Essential Medium (EMEM) supplemented with 2 mM Glutamine and 5% fetal bovine serum (FBS). During hPIV-1 infection, MA104 cells were maintained in EMEM supplemented with 2 mM Glutamine and 1.2% TrypLE Select (EMEM_INF_). hPIV-1 (strain C35) was obtained from the American Type Culture Collection and virus stock was amplified in MA104 cells at 35 °C, 5% CO_2_ in EMEM_INF_. The harvested virus was either stored at −80 °C for in situ ELISA, concentrated in Amicon Ultra-100 kDa spin-columns (Merck, Darmstadt, Germany) and stored at 4 °C for haemagglutination inhibition assays or sucrose purified as previously described [13] and stored at 4 °C for ^1^H NMR experiments and neuraminidase inhibition assays.

### 2.3. Neuraminidase Inhibition Assay

The neuraminidase inhibition potencies of compounds **2** and **3** were assessed using a method adapted from Potier et al. [20]. Sucrose purified virus was diluted in reaction buffer (50 mM NaOAc, 5 mM CaCl_2_, pH 5) to a concentration yielding at least 5 times the measured fluorescent signal background. NI assays were performed as previously described, with inhibitor concentrations ranging from 1 mM to 1 nM [13].

### 2.4. Haemagglutination Inhibition Assay

Compounds **2** and **3** were assessed in haemagglutination inhibition assays with human red blood cells using a previously reported procedure [13]. The assay was conducted at room temperature (22 °C) with inhibitor dilutions ranging from 50 μM to 12 nM.

### 2.5. In Situ ELISA

This technique has been modified from previous inhibition growth assays performed with hPIV-3 [17,19,21]. MA104 cells were plated in a 96-well plate to reach confluency after 24 h. 500 focus-forming units (FFU) of hPIV-1 were diluted in 50 μL of EMEM_INF_, and applied on the cell monolayer for incubation at 35 °C, 5% CO_2_ for 1 h with gentle agitation every 15 min. The virus inoculum was removed and replaced with 100 μL of EMEM_INF_ with appropriate compound concentrations (400 μM to 4 nM). Each assay was performed in triplicates. The plate was then incubated for 48 h at 35 °C, 5% CO_2_. The cells were fixed and the virus inactivated by adding 100 μL of 6.4% paraformaldehyde solution diluted in PBS to each well for 20 min. After washing with PBS, permeabilisation of the cells and inactivation of endogenous peroxydases was performed by adding 50 μL of a PBS solution containing 0.3% H_2_O_2_ and 1% IGEPAL for 20 min. The cells were washed with PBS and incubated with a mouse monoclonal IgG anti-hPIV-1 HN (LSBio, cat. LS-C74109, Seattle, WA, USA), at 0.5 μg/mL in 5% milk-PBS, for 45 min at 37 °C. After washing with 0.02% Tween20-PBS, cells were incubated with a goat polyclonal IgG anti-mouse IgG(H+L) HRP-conjugated (Biorad, Hercules, CA, USA) at a 1:2000 dilution in 5% milk-PBS for 45 min at 37 °C. Following a final wash with 0.02% Tween20-PBS, 3,3′,5,5′tetramethylbenzidine (TMB) substrate (BD OptEIA, BD Biosciences, North Ryde, Australia) was added to each well and incubated at room temperature (RT) for 5 min or until the background signal started to rise. The reaction was stopped by adding 50 µL of 0.6 M of H_2_SO_4_. The absorbance was read at 450 nm on a Biorad xMark™ Microplate Absorbance Spectrophotometer. The absorbance values were normalised to the negative control (non-infected cells) and expressed as a percentage of inhibition of hPIV-1 growth. The IC_50_ value was defined as the average concentration of a compound leading to a 50% reduction of hPIV-1 infected cells compared to a non-treated, infected well.

### 2.6. Recombinant hPIV-1 HN Expression and Purification

Recombinant baculovirus containing the coding sequence for the ectodomain of hPIV-1 HN strain C-35 (amino acids 137 to 575 of NCBI GeneBank entry AB542810) was obtained using the Bac-to-Bac^®^ expression system (Invitrogen, Carlsbad, CA, USA). The hPIV-1 HN was codon optimised for expression in *Spodoptera frugiperda*, amplified by PCR and inserted in the pFastBac™/HBM-TOPO. The plasmid contains the honeybee melittin secretion signal and a 6× Histidine C-terminal tag for purification. The recombinant bacmid was obtained following the manufacturer instructions and transfected in Sf9 cells (Invitrogen) to generate a baculovirus containing the hPIV-1 HN gene. TriEx™ Sf9 cells (Novagen, Madison, WI, USA) cultured in Insect-XPRESS protein free medium (Lonza, Basel, Switzerland) were infected with the recombinant baculovirus and incubated at 27 °C in a shaking incubator (100 rpm) for 96 h. The cell culture supernatant containing the HN protein was clarified by a centrifugation at 3000× *g* for 30 min. The protein was purified from the supernatant on a HisTrap excel 5 mL column (GE Healthcare Life Sciences, Little Chalfont, UK) following the manufacturer instructions. The bound HN was eluted with a 500 mM imidazole solution and the protein-containing fractions were pooled. Fractions from the affinity purification were further purified by size exclusion chromatography on a Superdex 200 pg column mounted on an ÄKTA pure L system. Protein-containing fractions were monitored by measuring the UV absorbance at 280 nm and the presence of HN was assessed by SDS-PAGE, Western blot (mouse IgG anti-HN, LSBio), and NA assay. The purified protein was concentrated by ultrafiltration on Amicon Ultra-10 kDa spin-column and stored at 4 °C.

### 2.7. ^1^H NMR Experiments

All NMR experiments were performed on a 600 MHz NMR spectrometer (Bruker, Billerica, MA, USA) equipped with a 5 mm Triple Resonance Inverse (TXI) probe with triple axis gradients. Recombinant hPIV-1 HN was buffer exchanged against deuterated 50 mM NaOAc, 5 mM CaCl_2_, pD 5 using Amicon Ultra 10 kDa filtering units (Merck). For each experiment, 5 μg of HN were incubated with 50 μM of compound **2** or **3**, or D_2_O for 1 h at 37 °C in a final volume of 50 μL. The protein was washed thrice with 450 μL of deuterated buffer using the Amicon spin-column to dilute compounds **2** and **3** to a final concentration of 12.5 nM. In each tube, 5 mM of 4-methylumbelliferyl α-d-*N*-acetylneuraminide were added to the protein in a total volume of 200 μL. ^1^H NMR spectra with 32 scans were acquired regularly over a 12 h period at 298 K. The tubes were incubated in a 37 °C water bath between measurements in the 600 MHz NMR spectrometer.

## 3. Results

### 3.1. hPIV-1 HN Neuraminidase Activity Characterisation and Blockade

To understand the mechanism underlying hPIV-1 HN neuraminidase glycohydrolase activity, the enzymatic activity of the viral protein was monitored using 4-methylumbelliferyl α-d-*N*-acetylneuraminide (MUN) as a sialidase substrate. This substrate contains a fluorescent component (4-methylumbelliferone, MU) conjugated to *N*-acetylneuraminic acid (Neu5Ac) that acts as a fluorescence quencher, when conjugated to the fluorophore. Upon hydrolysis of MUN by a neuraminidase activity, such as in hPIV-1 HN, free Neu5Ac and fluorescent 4-methylumbelliferone are produced. This reaction can be monitored using ^1^H NMR spectroscopy by measuring the gradual increase of the intensity of H3_ax_ and H3_eq_ proton peaks obtained after the release of either α- or β-Neu5Ac, and depending on the initial stereochemistry around the anomeric centre (C-2) of the released product.

To measure the turnover of hPIV-1 HN substrate hydrolysis, we monitored the cleavage of MUN by hPIV-1 HN using 5 μg of hPIV-1 HN recombinant protein and 5 mM of MUN in 200 μL of reaction buffer, at 37 °C for 60 min. In the absence of inhibitors, complete hydrolysis of MUN occurred within 15 min, where the neuraminic acid H3_eq_ peak associated with MUN disappeared and only peaks for H3_ax_ and H3_eq_ corresponding to released Neu5Ac α-anomer were observed. The released Neu5Ac α-anomer was found to mutarotate, as expected, to β-Neu5Ac after 60 min of incubation, where the intensities of the H3 peaks that correspond to the α-anomer started to decrease, while the intensities of the H3 peaks of the β-anomer started to increase (see Supplementary Figure S1 online). All together, these results revealed that hPIV-1 HN is a retaining glycohydrolase that initially maintained its substrate anomeric centre α-configuration, as it has been previously reported for several other sialidases [14,16,17,22].

**Figure 2:**
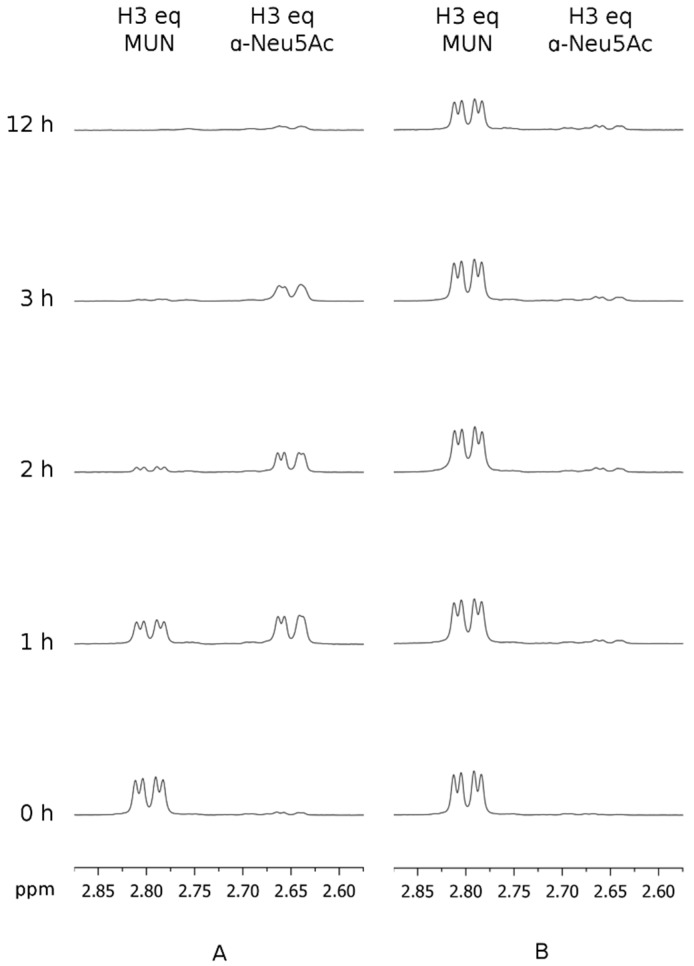
Time course study of the blockade of human parainfluenza virus-1 haemagglutinin-neuraminidase (hPIV-1 HN) neuraminidase activity by compounds **2** and **3.** The hydrolysis of 4-methylumbelliferyl α-d-*N*-acetylneuraminide (MUN) by hPIV-1 HN, pre-incubated with either compound **2** or **3** (50 μM), was monitored by ^1^H NMR spectroscopy over 12 h at 37 °C. (**A**) 5 μg of HN preincubated with **2**, in the presence of 5 mM of MUN. (**B**) 5 μg of HN preincubated with **3**, in the presence of 5 mM of MUN. **eq**: equatorial.

Subsequently, the reference hPIV-1 HN inhibitor BCX 2798 (**2**) and its 2α,3β-difluoro derivative (**3**) were used to further investigate the catalytic mechanism of hPIV-1 HN. The relative capacities of compounds **2** and **3** to block hPIV-1 HN neuraminidase activity were determined by measuring the hPIV1 HN-hydrolysis kinetics of MUN in the presence of these inhibitors. Thus, 5 μg of purified hPIV-1 HN protein was pre-incubated with 50 μM of inhibitor **2** or **3** for 1 h at 25 °C and subsequently washed with reaction buffer using a spin-column until the remaining inhibitor concentration was theoretically under 12.5 nM in the reaction volume. At that final concentration, both compounds **2** and **3** are considered to have null or insignificant inhibitory effect towards hPIV-1 HN neuraminidase activity. The neuraminidase substrate MUN was then added at a final concentration of 5 mM and its hydrolysis was assessed over 12 h while incubated at 37 °C. In this experiment, we observed that inhibitor **2** was able to slow down the hydrolysis of MUN with the endpoint of the reaction occurring between 2 and 3 h after the addition of MUN (Figure 2A). In contrast, when hPIV-1 HN was pre-incubated with compound **3**, only a trace amount of α-Neu5Ac was released from MUN over a period of 12 h, which reflects a prolonged blockade of hPIV-1 HN NA function (Figure 2B). As shown in Figure 2B, a decrease in the intensity of the H3_eq_ peak of MUN was observed after 12 h, this however could be attributed to the acidic hydrolysis of the compound under assay conditions rather than being the result of enzymatic hydrolysis. To confirm this notion, the hydrolysis of 5 mM of MUN alone was monitored for 12 h in the same acidic conditions. As expected, a comparable decrease in the intensity of H3_eq_ peak of MUN was observed, which means that this observed reduction in MUN concentration was not the result of enzymatic hydrolysis (see Supplementary Figure S2 online).

### 3.2. Inhibition of hPIV-1 HN Haemagglutination and Neuraminidase Functions

**Figure 3:**
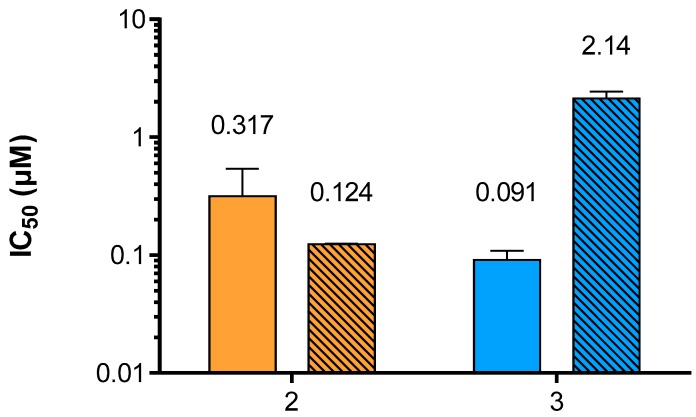
Evaluation of compounds **2** and **3** potencies to inhibit hPIV-1 HN neuraminidase and haemagglutinin functions. Inhibition of hPIV-1 HN neuraminidase (plain) and haemagglutinin (stripes) functions by inhibitors **2** and **3**. The values presented are the mean of 3 independent experiments; the calculated standard deviation (SD) is represented by the error bars.

Neu5Ac2en-based inhibitors target the primary sialic acid-binding site of hPIV-1 HN, which is also the site for its neuraminidase function. While Neu5Ac2en (**1**) is a relatively poor inhibitor of hPIV-1 HN haemagglutination and neuraminidase functions, BCX 2798 (**2**) has been shown to inhibit these functions at sub-micromolar levels [13]. The neuraminidase inhibition (NI) potency of compound **3** was evaluated using a previously described method [12] adapted from Potier et al. that measures the relative fluorescence of MU released from the cleavage of MUN by hPIV-1 HN in the presence or absence of inhibitor (Figure 3). In this assay, a 3.5-fold potency increase was observed for inhibitor **3** when compared to **2**, with NI IC_50_ values of 90.9 nM and 317 nM, respectively. Considering HN’s multifunctionality, it was also important to assess the ability of compound **3** to inhibit the hPIV-1 HN haemagglutination function (HI) in order to evaluate the impact of difluorination of BCX 2798 on its dual inhibitory capacity. Therefore, the inhibition of hPIV-1 HN haemagglutination of human red blood cells (RBC) was measured at 22 °C and pH 7.4, using a previously reported procedure [13]. Interestingly, we observed a decrease in HI potency for **3** with an IC_50_ value of 2.14 μM compared to 124 nM for **2** (Figure 3). This is, however, consistent with our previous observations for hPIV-3 HN, where weaker inhibition of the hPIV-3 HN haemagglutinin function was observed for the difluorosialic acid compound relative to its Neu2en counterpart [21].

### 3.3. In Vitro Viral Replication Inhibition

**Figure 4:**
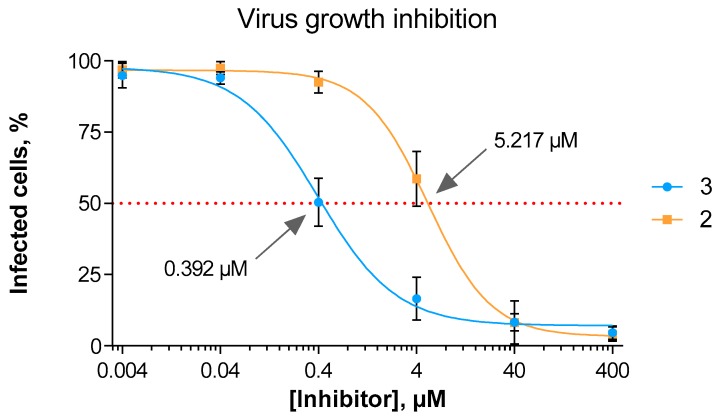
hPIV-1 growth inhibition by compounds **2** and **3.** The data represents the mean of three independent experiments; the error bars represent the calculated standard deviation (SD).

Demonstrating inhibitory potency of compound **3** on both hPIV-1 HN neuraminidase and haemagglutinin functions, we felt it important to assess inhibitor **3** for its capacity to block virus proliferation in vitro. Knowing that inhibitors **2** and **3** blocked haemagglutination and neuraminidase activity to different extents we now wanted to assess if these differences contributed to an overall reduction of viral replication. Thus, using an in situ ELISA method, we assessed the potency of compound **3** compared to the reference hPIV-1 HN inhibitor **2** in reducing the overall spread of hPIV-1 after a MA104 cell monolayer was infected with a low multiplicity of infection (Figure 4). Noteworthy, we observed a 13-fold increase in potency of compound **3** relative to **2**, with determined IC_50_ values of 0.39 μM and 5.22 μM, respectively.

## 4. Discussion

**Figure 5:**
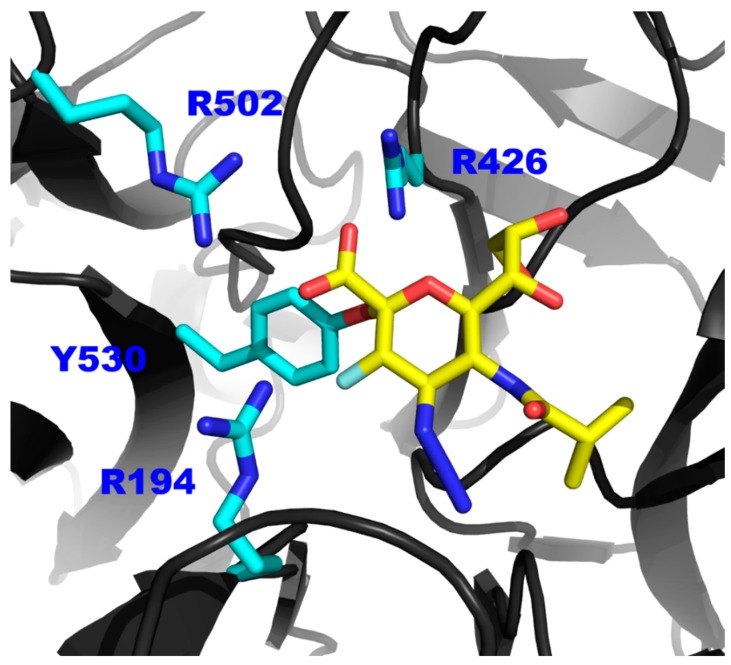
The proposed model of the interaction between inhibitor **3** and the Tyr530 of hPIV-1 HN. Representation of a hPIV-1 homology model (based on hPIV-3 co-crystal structure in complex with **3,** PDB ID: 4XJR) [13] in complex with inhibitor **3** (yellow sticks). Side chains of the tri-arginyl cluster (R194, R426 and R502) and the key catalytic amino acid Tyr530 are shown in blue sticks.

The structural similarity between all sialidase active sites suggests a similar catalytic mechanism. The catalytic mechanism of a range of sialidases, including the closely related hPIV-3 HN neuraminidase, has been investigated and confirmed this mechanistic similarity [14,15,16,17]. We previously reported that the 2α,3β-difluorinated compound **3** was able to covalently link to the catalytic Tyr530 residue within the hPIV-3 HN active site, forming a covalent adduct and consequently inducing a long-lasting hPIV-3 NA inhibition [17]. In the absence of a hPIV-1 HN crystal structure, we relied on ^1^H NMR experiments to evaluate the capacity of compound **3** to produce a similar prolonged inhibition of the neuraminidase function of hPIV-1 HN. Our ^1^H NMR experiments demonstrated that inhibitor **3** successfully induced a prolonged inhibition for the hPIV-1 HN NA function (relative to compound **2**), similar to what was observed with hPIV-3 HN. In contrast, a continuous turnover of the substrate was monitored using the reference inhibitor **2** in the same experimental set-up. We propose that this prolonged inhibition is the result of a covalent adduct formation between **3** and the catalytic Tyr530 residue of hPIV-1 HN. This catalytic residue is anticipated to be involved in the catalytic binding mechanism of hPIV-1 HN in a similar fashion to hPIV-3 HN, as shown in Figure 5 using our previously generated homology model for hPIV-1 HN [13].

Furthermore, similar to its Neu2en counterpart, the 2α,3β-difluoro derivative of BCX 2798 (**3**) is able to inhibit both hPIV-1 HN neuraminidase and haemagglutinin functions at nanomolar and micromolar levels, respectively. The functionalisation around the C-2 and C-3 of compound **3** resulted in a slight increase in its inhibitory potency against the HN’s neuraminidase activity and a decrease in its potency against the HN’s haemagglutinin activity compared to its Neu2en analogue **2**. However, viral replication in the presence of compound **3** was strongly reduced as shown from the in situ ELISA testing, with a cell-based assay IC_50_ value reported, for the first time in the sub-micromolar range for a hPIV-1 inhibitor. This overall improvement at the cellular level can be explained by the ^1^H NMR analysis, which shows that inhibitor **3** is capable of blocking HN’s neuraminidase activity for at least 12 h. The dual functionality of hPIV HN protein and the fact that both receptor binding and cleaving functions reside in the same binding/catalytic domain [23] explains why a prolonged engagement of the inhibitor to this site would result in a more significant inhibition of viral replication. The prolonged blockade of the HN primary binding/catalytic site simply translates into a longer and more efficient interference with two stages of the viral life cycle: The release of virion progeny and most importantly the subsequent binding to host cells, as previously suggested for hPIV-3 HN [24]. 

In summary, the 2α,3β-difluoro-*N*-acylneuraminic acid **3** was utilised as a probe to study the catalytic mechanism of the hPIV-1 HN protein. The prolonged blockade of the HN’s neuraminidase activity suggests the potential formation of a covalent adduct between inhibitor **3** and a catalytic residue in the binding pocket. This residue is likely to be the key amino acid Tyr530, where a covalent adduct was confirmed between this residue and **3** in the closely related hPIV-3 HN protein [17]. This apparent covalent link between **3** and a catalytic residue in the binding pocket suggests that hPIV-1 HN protein follows a similar catalytic mechanism to other neuraminidases. In this common mechanism a two-step, double-displacement reaction involves covalent bond formation between a highly-conserved tyrosine residue (e.g., Tyr530) and C-2 of the bound sialic acid moiety.

In addition, we have also reported for the first time the use of 2α,3β-difluoro-*N*-acylneuraminic acid based inhibitors, exemplified by inhibitor **3**, as a novel category of potent mechanism-based inhibitors for hPIV-1 HN. In spite of only a slight improvement in NI potency combined with a decrease in HI potency of this inhibitor relative to its Neu2en counterpart (**2**), a significant overall improvement in viral replication blockade was observed. This is likely the result of a prolonged blockade of the catalytic/receptor binding pocket, that interferes with both functions of the HN protein, resulting in a significant decrease in both the release of virion progeny from the infected cell membrane and subsequent infection of new cells.

## Supplementary Materials

The following are available online at https://www.mdpi.com/1999-4915/11/5/417/s1, Figure S1: Time course study of the hydrolysis of MUN by hPIV-1 HN, Figure S2: Acidic hydrolysis of MUN over 12 hours.
