# Supplementary material for: Targeting Human Parainfluenza Virus Type-1 Haemagglutinin-Neuraminidase with Mechanism-Based Inhibitors
Source: Viruses. 2019 May 5;11(5):417. doi: 10.3390/v11050417 (PMC6563277; doi:10.3390/v11050417)

viruses

Supplementary Information

Targeting human parainfluenza virus type-1 haemagglutinin-neuraminidase with mechanism-based inhibitors

Tanguy Eveno[1], Larissa Dirr[1], Ibrahim M. El-Deeb[1+], Patrice Guillon[1+] and Mark von Itzstein[1*+]

[1] Institute for Glycomics, Griffith University, Gold Coast Campus, Queensland, 4222, Australia.

* Correspondence and requests for materials should be addressed to m.vonitzstein@griffith.edu.au

[+] Ibrahim M. El-Deeb, Patrice Guillon and Mark von Itzstein jointly supervised this work.

Supplementary figure legends

Supplementary Figure S1. Time course study of the hydrolysis of MUN by hPIV-1 HN. The hydrolysis of MUN and the mutarotation of the released α-Neu5Ac into β-Neu5Ac were monitored by ^{1}H NMR spectroscopy. The reaction contained 5 µg of HN, 5 mM of MUN in a final volume of 200 µL of 50 mM NaOAc, 5 mM $CaCl_2$, pD 5 and was incubated at 25 °C for 1 hour. eq: equatorial, ax: axial. The other signals at ~2.35 and 1.95 ppm are associated with the CH_3 group of MUN and the acetamido moiety of α,β-Neu5Ac, respectively.

Supplementary Figure S2. Acidic hydrolysis of MUN over 12 hours. The acidic hydrolysis of MUN in the reaction buffer (50 mM NaOAc, 5 mM $CaCl_2$, pD 5) was monitored by ^{1}H NMR spectroscopy. The reaction contained 5 mM of MUN in 200 µL of reaction buffer. eq: equatorial, ax: axial. The other signals at ~2.35 and 1.95 ppm are associated with the CH_3 group of MUN and the acetamido moiety of α,β-Neu5Ac, respectively.

Supplementary Figure S1

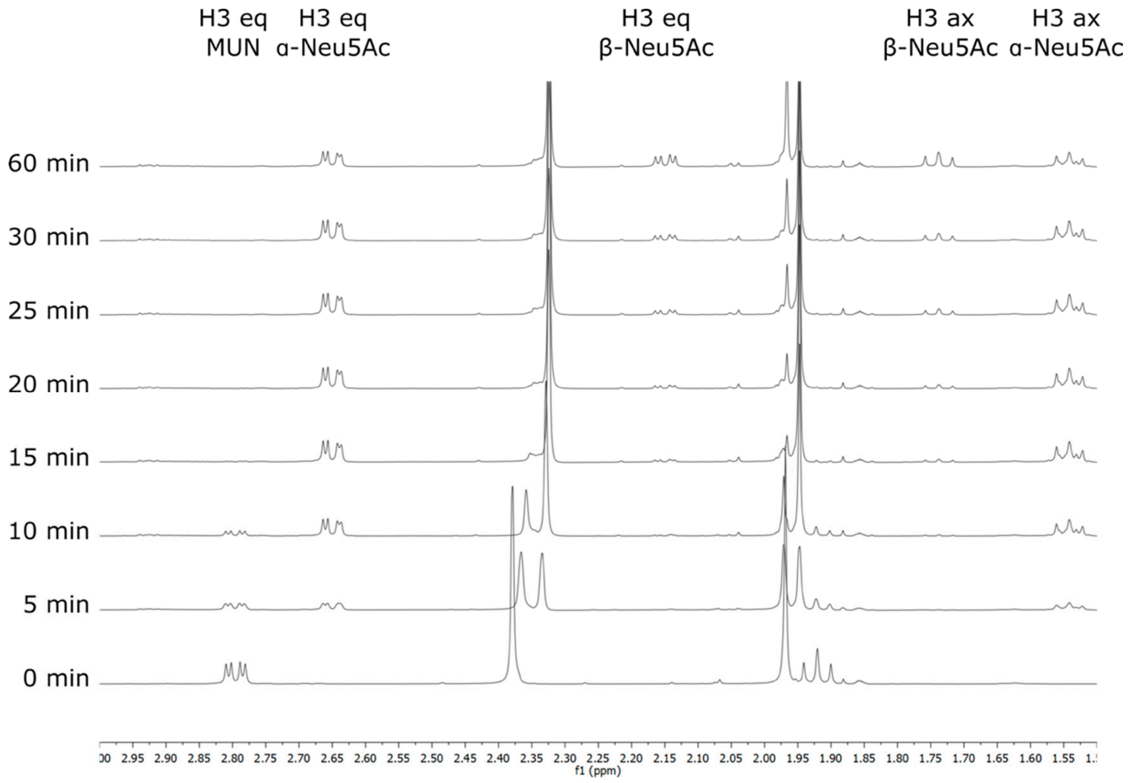

Supplementary Figure S2

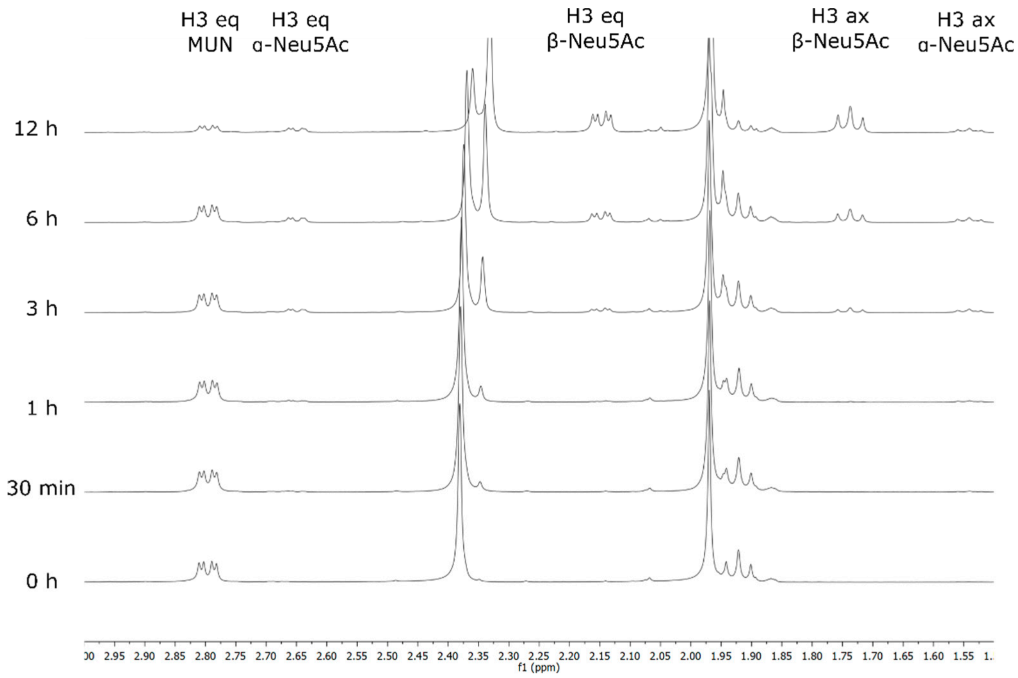

Supplement: Supplementary file 1 [file viruses-11-00417-s001.pdf]
